# Hearing loss in Iranian thalassemia major patients treated with deferoxamine: A systematic review and meta-analysis 

**DOI:** 10.22088/cjim.8.4.239

**Published:** 2017

**Authors:** Gholamreza Badfar, Akram Mansouri, Masoumeh Shohani, Hamid Karimi, Zahra Khalighi, Shoboo Rahmati, Ali Delpisheh, Yousef Veisani, Ali Soleymani, Milad Azami

**Affiliations:** 1 Department of Pediatrics, Behbahan Faculty of Medical Sciences, Behbahan, Iran; 2 Nursing Care Research Center in Chronic Disease, School of Nursing and Midwifery, Ahvaz Jundishapour University of Medical Sciences, Ahvaz, Iran; 3 Department of Nursing, Faculty of Allied Medical Sciences, Ilam University of Medical Sciences, Ilam, Iran.; 4 Department of Endocrinology, Faculty of Medicine, Dezful University of Medical Sciences, Dezful, Iran; 5 Biotechnology and Medicinal Plants Research Center, Ilam University of Medical Sciences, Ilam, Iran; 6 Student Research Committee, Ilam University of Medical Sciences, Ilam, Iran; 7 Department of Clinical Epidemiology, Ilam University of Medical Sciences, Ilam, Iran; 8 Psychosocial Injuries Research Center, Ilam University of Medical Sciences, Ilam, Iran; 9 Faculty of Medicine, Dezful University of Medical Sciences, Dezful, Iran

**Keywords:** Hearing loss, Thalassemia major, Deferoxamine, Systematic review, Meta-analysis, Iran

## Abstract

**Background:**

Hearing disorders are reported in thalassemia patients treated with deferoxamine. This study aimed to assess hearing loss in Iranian thalassemia major patients treated with deferoxamine.

**Methods:**

This review article was designed based on PRISMA guidelines. To review the literature, two researchers studied national and international databases including Iranmedex, Magiran, Medlib, SID, Scopus, PubMed, Science Direct, Web of Science and Google Scholar without time limit until May 2017. Cochran's Q test and I^2^ index were used to assess the heterogeneity of the studies. The data were analyzed using Comprehensive Meta-Analysis software version 2 and p<0.05 was considered significant.

**Results:**

A total of 17 articles involving 1,835 Iranian thalassemia major patients treated with deferoxamine were included in the meta-analysis. The overall prevalence of hearing loss was estimated 27.3% (95% confidence intervals (CI): 19-37.6). The prevalence of sensorineural, conductive and mixed hearing loss was estimated 10.6% (95% CI: 5.7-18.8), 14.6% (95% CI: 10.5-20.6) and 9.1% (95% CI: 5.6-14.6), respectively. No significant differences were noted regarding the relationship hearing loss and mean serum ferritin (P=0.29) and average daily deferoxamine (P=0.30). Meta-regression model showed an increased significance in the prevalence of hearing loss based on the year of studies (p<0.0001).

**Conclusions:**

There is a high prevalence of hearing loss in Iranian thalassemia major patients treated with deferoxamine. Therefore, periodic hearing assessments and regular check-ups after the initiation of chelation therapy are necessary.

Thalassemia is a common disorder in Southeast Asia caused by genetic defects in the synthesis of hemoglobin chain leading to chronic hypochromic microcytic anemia (1). These patients can be treated by receiving regular monthly blood transfusions that decreases the acute symptoms of the disease. On the other hand, frequent blood transfusions can lead to hemosiderosis and tissue dysfunction including hepatic, cardiac and endocrine complications (2-5). These patients die in the second decade of life due to the mentioned complications. But today, the survival rate has greatly improved because of the advances in therapies, especially after the chelation therapy. A relatively long lifetime, is expected for the patients with appropriate treatment (6). Despite the benefits of chelation therapy in these patients, complications are unavoidable. Hearing disorders are reported in thalassemia patients treated with deferoxamine (DFO) (7-8). 

A simple review of the documents indicated that the prevalence of hearing loss in patients with thalassemia major treated with DFO varied in Iran (9-13) and now a meta-analysis is necessary to summarize the results and provide an overview of the problem. Meta-analysis is a method of collecting and analyzing the results of several studies with the same goal and providing a reliable estimation of the effect of some medical interventions or observations (14-15). Based on this method, as the number of samples increased, the range of changes and possibilities decreased and the significance of the statistical results increased. This method is more reliable due to the certain conditions of meta-analysis (16-17). This study aimed to assess hearing loss in patients with thalassemia major treated with DFO in Iran using the meta-analysis method.

## Methods

**Study protocol** **: **This review study was designed based on the recommendations of PRISMA (18). To avoid bias in this study, all procedures were done by two researchers independently. Group discussion was used in case of divergence in the results.

**Inclusion and exclusion criteria:** The main inclusion criteria were all the epidemiological studies that assessed hearing loss in patients with thalassemia major. Exclusion criteria included: (1) non-random sample size for prevalence estimate; (2) non-Iranian patients; (3) patients not treated with DFO; (4) being irrelevant to the topic; and (5) review studies, letters to the editor, case reports.

**Search strategy: **To review the literature, researchers studied the national and international databases, including Iranmedex, Magiran, Medlib, SID (Scientific Information Database), Scopus, PubMed, Science Direct, Web of Science and Google Scholar without time limit until May 2017. To maximize the comprehensiveness of the research in the databases, we used MeSH keywords including Prevalence, Epidemiology, Hearing Loss, Sensorineural, Drug-Related Side Effects, Deferoxamine, Ferritin, Iron Overload, Chelation Therapy, Thalassemia and Iran and then combined our search with operators AND/OR in English databases. PubMed combination search is shown in appendix 1. References were also evaluated to find further studies.

**Quality assessment: **Researchers examined the selected articles using STROBE checklist (19), which is a standard and an international checklist for the quality assessment of the studies. The authors have adopted a simple method for scoring. Each question was scored between 0-2 points. The maximum point was 44 and the minimum acceptable point for inclusion in the meta-analysis was 16. The researchers focused on a group discussion in case of discrepancies. 

**Data Extraction: **To reduce the errors in data collection, data extraction was done using a data extraction form including authors’ name, data publication, year of study, number of participants, the overall prevalence of hearing loss, prevalence of conductive hearing loss, prevalence of sensorineural hearing loss, dB HL (decibels hearing level), the association between hearing loss and ferritin (mean serum ferritin in case and control groups and number of patients with hearing loss; equal or above 3000 vs. [versus] lower than 3000 ng/ml), the association between hearing loss and age (number of patients with hearing loss; equal or above 10 years-old vs. below 10) and for the association between hearing loss and average daily DFO use (mean daily DFO use in case and control groups). we asked specific questions or ambiguities from the authors of the articles via email.

**Statistical analysis: **The binomial distribution was used to calculate the variance of each study for the prevalence of hearing loss in patients with thalassemia major. To study the relationship between hearing loss and ferritin, average daily DFO use and age, odds ratio (OR) and the standardized mean difference (SMD) were used. Cochran's Q test and I^2^ index were used to assess heterogeneity of the studies. In this study, the heterogeneity was found to be 92.5% for the prevalence of hearing loss, which was a high heterogeneity (I^2^ index less than 25% is considered as low heterogeneity, between 25-75% is moderate heterogeneity and more than 75% is high heterogeneity). So, the random effects model was used in the meta-analysis. 

To investigate the effect of ferritin (≥3000 vs. <3000) on hearing loss, random effects model was used due to high heterogeneity and for other variables, fixed effects model was used due to low heterogeneity (20-21). To find the source of high heterogeneity for the prevalence of hearing loss, subgroup analysis was conducted based on province and thresholds of sound intensity in audiometry. To investigate the relationship between hearing loss and the year of studies, meta-regression model was used. The data were analyzed using Comprehensive Meta-Analysis software Version 2 and p<0.05 was considered significant.

## Results

**Figure1:**
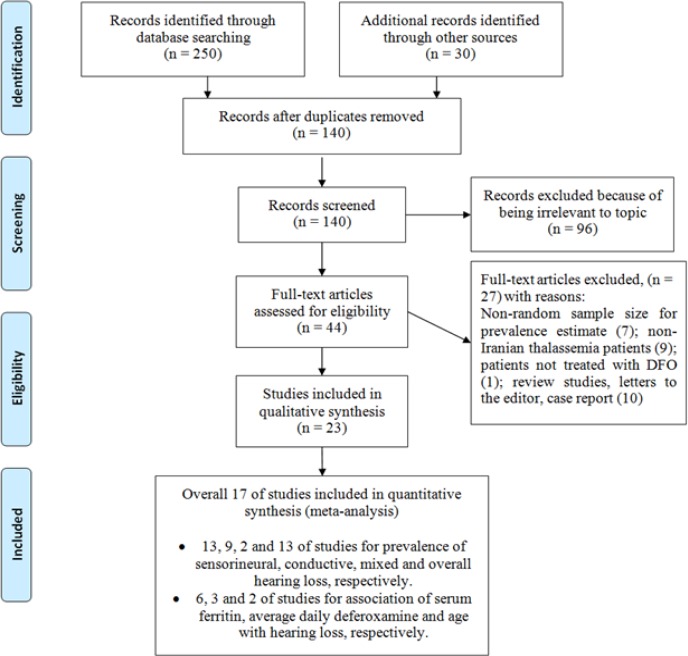
A flowchart of the literature searches for the systematic review of studies

**Search** **results: **280 relevant studies were found in the initial systematic review by two authors and 140 studies were excluded due to duplication. After screening the titles and abstracts, 96 studies were excluded because of their irrelevance to the topic. Finally, 17 studies with appropriate quality entered the meta-analysis (figure 1).

**Characteristics of the included studies: **The participants of this study were 1,835 Iranian thalassemia major patients treated with DFO.

The mean age of the participants was 12.5 yr (95% confidence intervals (CI): 10.0-14.9). Details of the studies that entered the meta-analysis are shown in table 1.

**Figure 2:**
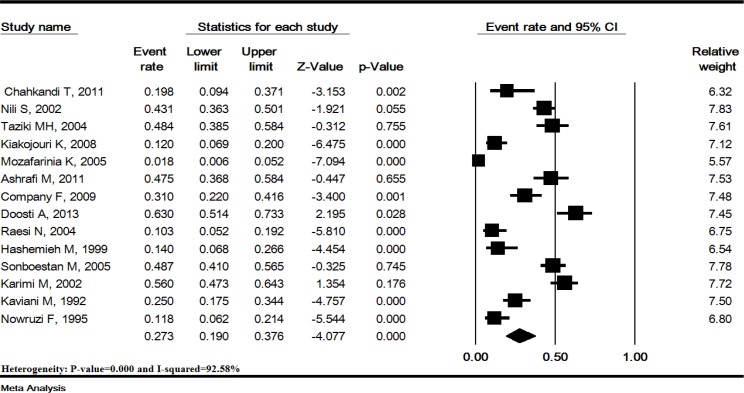
Prevalence of hearing loss in Iranian thalassemia major patients treated with deferoxamine. Random effects model.

**Overall prevalence of hearing loss: **The rate heterogeneity in this study was 92.5% (p<0.0001). In 14 studies including1,422 Iranian thalassemia major patients treated with DFO, the overall prevalence of hearing loss was estimated 27.3% (95% CI: 19-37.6). The lowest prevalence of hearing loss was reported in a study conducted in Kerman (1.8%) while the highest prevalence was reported in a study conducted in Yazd (63%) (figure 2).

**Figure 3:**
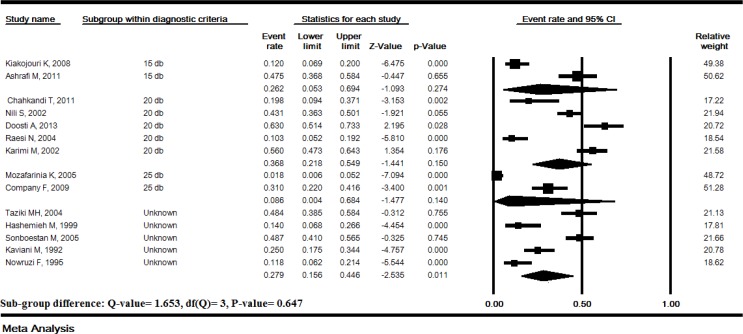
Subgroup analysis of hearing loss prevalence based on thresholds of sound intensity in Iranian thalassemia major patients treated with deferoxamine. Random effects model

**Subgroup analysis** **of** **hearing loss** **prevalence based on** **threshold** **s: **The prevalence of hearing loss based on the thresholds 15, 20 and 25 dB HL of sound intensity in audiometry was 26.2% (95% CI: 5.3-69.4), 36.8% (95% CI: 21.8-54.6) and 8.6% (95% CI: 0.4-68.4), respectively and the subgroup difference was not significant (P=0.64) (figure 3).

**Figure 4:**
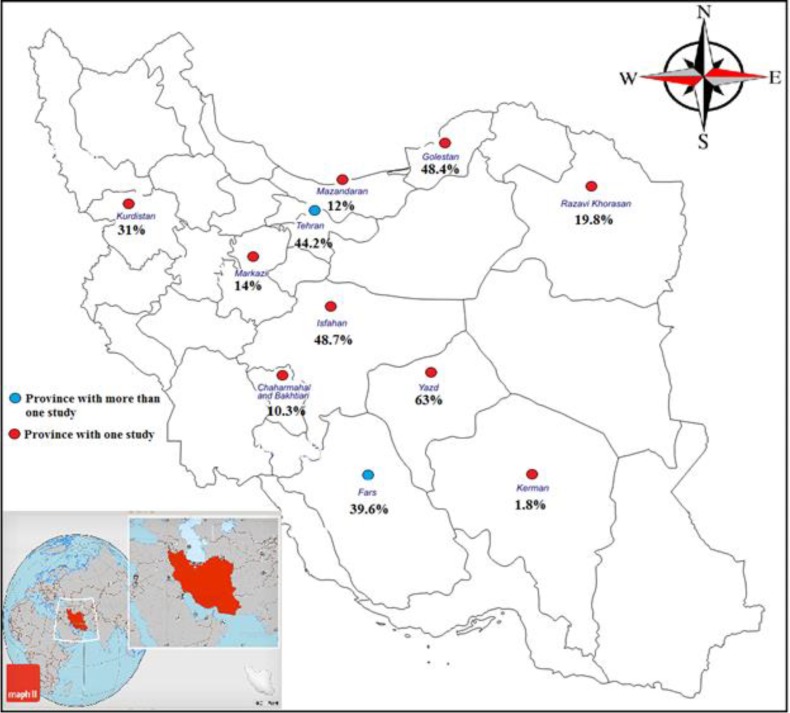
Geographical distribution of prevalence of hearing loss in Iranian thalassemia major patients treated with deferoxamine

**Subgroup analysis** **of** **hearing loss** **prevalence based on provinces: **The prevalence of hearing loss based on provinces was shown in figure 4 and the subgroup difference was significant (p<0.0001).

**Table 1:** Summary of the included studies

**Prevalence of hearing loss (%)**				**Age ** **Mean±SD**	**Sample size**	**Year**	**Place**	**The first author, publication date**	**Ref**
**Overall**	**Mixed**	**Conductive**	**Sensorineural**						
19.8		9.4	9.4	9.3±1.4	32	2007	Birjand	Chahkandi T, 2011	9
43.1		16	4.6		195	2002	Tehran	Nili S, 2002	10
48.4					95	2001	Gorgan	Taziki MH, 2004	11
12		12			100	2006	Babol	Kiakojouri K, 2008	12
1.8					178	2003	Kerman	Mozafarinia K, 2005	13
47.5	8.75	17.5	21.2	14.2±2.3	80	2009	Tehran	Ashrafi M, 2011	22
30.9	9.5	9.5	11.9	12.8±5.7	84	2007	Sanandaj	Company F, 2009	23
63		8.2	54.8		73	2010	Yazd	Doosti A, 2013	24
10.3		10.3	0		78	2001	Shahrkord	Raesi N, 2004	25
14			14	10.0±2.5	50	1998	Arak	Hashemieh M, 1999	26
			3.5		293	2006	Shiraz	Faramarzi A, 2010	27
48.7					156	2003	Isfahan	Sonboestan M, 2005	28
56		32.3	11.7	14.4±5.0	128	2000	Shiraz	Karimi M, 2002	29
			7.4	14.3±5.1	67	2006	Tehran	Shamsian B.B, 2008	8
			5.7		53	2006	Tehran	Azizi G, 2008	30
25		20	5		100	1991	Shiraz	Kaviani M, 1992	31
			11.8		73	1994	Babol	Nowruzi F, 1995	32

**Figure 5:**
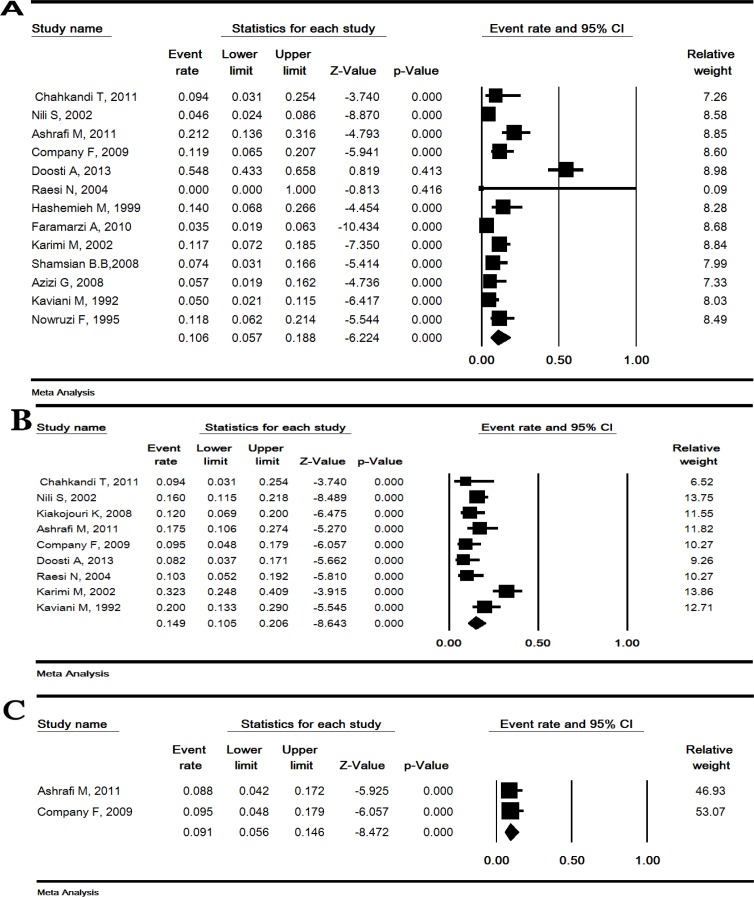
Prevalence of sensorineural (A), conductive (B) and mixed (C) hearing loss in Iranian thalassemia major patients treated with deferoxamine. Random effects model

**Prevalence of sensorineural, conductive and mixed hearing loss: **The prevalence of sensorineural, conductive and mixed hearing loss was estimated 10.6% (95% CI: 5.7-18.8), 14.6% (95% CI: 10.5-20.6) and 9.1% (95% CI: 5.6-14.6), respectively (figure 5). 

**The relationship between hearing loss and age, serum ferritin, and average daily dose of deferoxamine:**

**Figure 6:**
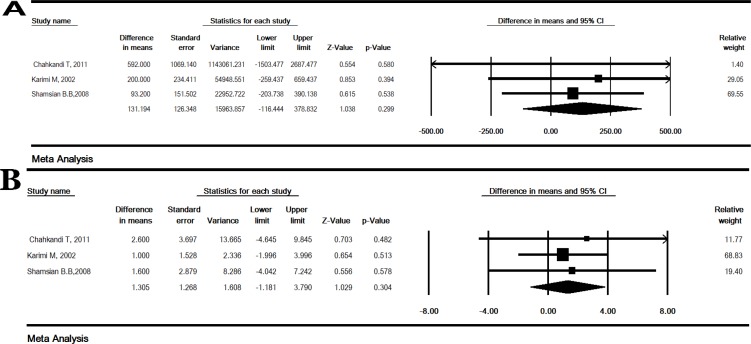
The the standardized mean difference for the mean serum ferritin (A) and average daily deferoxamine use (B) in Iranian thalassemia major patients treated with deferoxamine. Fixed effects model

The OR for the relationship between age (≥10 vs. <10 years) and hearing loss was 1.93 (95% CI: 1.24-3.02) and was statistically significant (p=0.004) (table 2). The relationship between serum ferritin (≥3000 vs. <3000 ng/ml) and hearing loss was estimated and OR was 3.34 (95% CI: 1.53-7.27), which was significant (p=0.002) (table 2). But this relationship between mean serum ferritin in patients with hearing loss versus without hearing loss was not significant (SMD: 131.19 [95% CI: -116.44 to 378.83], P=0.29) (figure 6 A). The SMD for the relationship of average daily DFO and hearing loss was 1.30 (95% CI: -1.18 to 3.79) and was not significant (P=0.30) (figure 6 B).

**Figure 7.**
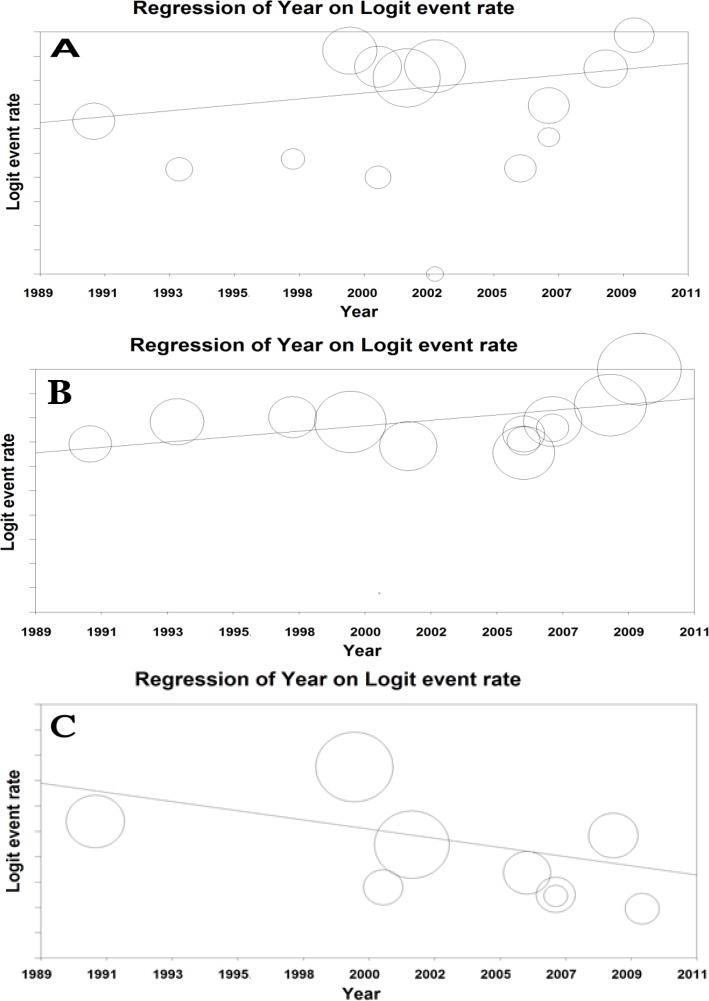
Meta-regression between the prevalence of overall (A), sensorineural (B) and conductive (C) hearing loss in Iranian patients with thalassemia major and year of studies. Larger circles indicate the larger sample size

**Meta-regression model: **Meta-regression model was used to assess the relationship between sensorineural, conductive and overall prevalence of hearing loss and year of studies and the p-value was <0.0001, which was statistically significant (figure 7).

**Table 2:** The association between age and serum ferritin with hearing loss in Iranian thalassemia major patients treated with deferoxamine

**P-Value**	**95% CI** c	**OR** b	**Heterogeneity**		**Without hearing loss** **(N**^a^**)**		**With hearing loss** **(N**^a^**)**		**Studies (N** a **)**	**Variable**
			**P** **-Value**	**I** ^2^	**All**	**Event**	**All**	**Event**		
0.004	1.24-3.02	1.93	0.81	0	80	29	82	58	2	Age ≥10 vs. <10 years
0.002	1.53-7.27	3.34	0.19	38.16	181	26	97	46	3	Ferritin ≥3000 vs. <3000 ng/ml

a Number;

b odds ratio;

c confidence interval

## Discussion

The present study is the first systematic review and meta-analysis about the prevalence of hearing loss in patients with thalassemia major treated with DFO in Iran. The overall prevalence of hearing loss was estimated 27.3% (19%-37.6%). The prevalence of sensorineural, conductive and mixed hearing loss was estimated 10.6%, 14.6% and 9.1%, respectively. The subgroup analysis of hearing loss prevalence based on provinces and thresholds of sound intensity was conducted and the difference was statistically significant (p<0.01). In other countries, the prevalence of sensorineural and conductive hearing loss in patients with thalassemia major treated with DFO were reported to be 3.8 - 57% (33-38) and 4.5 - 59.2% (34, 39-40), respectively. The variety in the prevalence of hearing loss can be attributed to genetic factors, individual characteristics, DFO administration, the length of DFO administration and different follow-up periods in different countries.

According to thalassemia prevention program (Ministry of Health, 2000), there are 20,000 patients with thalassemia receiving blood transfusions and iron chelation in Iran. DFO and deferiprone are considered as iron chelators in Iran, while DFO is more common and deferiprone is less common due to the complications such as agranulocytosis and the need for the routine evaluation of white blood count (WBC) (8). In a systematic review among patients with thalassemia major in Iran, almost all patients were treated with iron chelation drugs, but only 55% of the patients were treated with regular (41), which can be attributed to glandular and cardiac complications in patients with thalassemia major in Iran (42-44). In this study, the difference is mean serum ferritin was not significant in patients with hearing loss and without hearing loss, but the relationship between serum ferritin and hearing impairment was significant (≥ 3000 vs. 3000 ng/ml). The reason for this significant relationship can be attributed to the invasive treatment of deferoxamine to reduce iron overload in patients with ferritin levels above 3000 ng/ml. However, the small number of studies involved in the meta-analysis process can be another reason, which needs to be considered. Moreover, the relationship between age (≥10 vs. <10 years) and hearing loss was demonstrated. Therefore, a thalassemia major patient treated with DFO older than 10 years was more exposed to hearing complications. The mechanism of neurotoxicity of DFO chelation is not completely specified yet (45). A high dosage of DFO may cause a high level of unbound drug and this may directly result in neurotoxicity or interaction with other very rare elements (46-47). Young age of starting treatment, the dose of drug administration, and serum ferritin may increase the risk for ototoxicity (45, 47). Sensorineural hearing loss is not directly associated with serum ferritin or dose of DFO. However, other factors including genetic and constitutional characteristics may be associated with sensorineural hearing loss (8). Porter et al. found that the high dose of DFO is associated with low serum ferritin level (<2000 ng/ml) and is a major risk factor for desferal ototoxicity (45). Ambrosetti et al. demonstrated that there is no significant relationship between hearing loss and age, serum ferritin level and therapeutic index (39). Considering the changes in the prevalence of hearing loss based on the year in the meta-regression, the value is increasing based on the years of study (1998-2010), which is significant?

The limitations of this study include: 1. Low sensitivity of national databases for combined search; 2. Failure to provide statistics on the prevalence of hearing loss according to gender; 3. The limitation of the number of studies regarding the relationship between hearing impairment and age, and the mean ferritin that may affect the results.

In conclusion, the prevalence of hearing loss in patients with thalassemia major treated with DFO in Iran is high. Potential lesions causing hearing impairment may be present in any part of the auditory pathway. Therefore, periodical hearing assessments and regular check-ups after onset of chelation therapy are necessary. 

## Conflict of interest:

No conflict of interest in this article.
